# Protein Degradation by Gammaherpesvirus RTAs: More Than Just Viral Transactivators

**DOI:** 10.3390/v15030730

**Published:** 2023-03-11

**Authors:** Lauren R. Combs, Jacob Combs, Robert McKenna, Zsolt Toth

**Affiliations:** 1 Department of Oral Biology, University of Florida College of Dentistry, 1395 Center Drive, Gainesville, FL 32610, USA; 2 Department of Biochemistry and Molecular Biology, University of Florida College of Medicine, 1200 Newell Drive, Gainesville, FL 32610, USA; 3 UF Genetics Institute, Gainesville, FL 32610, USA; 4 UF Health Cancer Center, Gainesville, FL 32610, USA

**Keywords:** KSHV, RTA, ubiquitination, protein degradation, gammaherpesviruses, E3 ubiquitin ligases

## Abstract

Kaposi’s sarcoma-associated herpesvirus (KSHV) is a member of the Gammaherpesvirus subfamily that encodes several viral proteins with intrinsic E3 ubiquitin ligase activity or the ability to hijack host E3 ubiquitin ligases to modulate the host’s immune response and to support the viral life cycle. This review focuses specifically on how the immediate-early KSHV protein RTA (replication and transcription activator) hijacks the host’s ubiquitin–proteasome pathway (UPP) to target cellular and viral factors for protein degradation to allow for robust lytic reactivation. Notably, RTA’s targets are either potent transcription repressors or they are activators of the innate and adaptive immune response, which block the lytic cycle of the virus. This review mainly focuses on what is currently known about the role of the E3 ubiquitin ligase activity of KSHV RTA in the regulation of the KSHV life cycle, but we will also discuss the potential role of other gammaherpesviral RTA homologs in UPP-mediated protein degradation.

## 1. Introduction

As obligate intracellular parasites, viruses need their host to survive and, as a result, they have adapted themselves to hijack, subvert, and circumvent the anti-viral safe-guards of the host; and herpesviruses are no exception [1]. Herpesviruses are large, enveloped, double-stranded DNA viruses that express several viral factors to avoid detection by the host’s immune system, thereby allowing them to establish lifelong infections. The Herpesviridae family is further divided into three subfamilies: Alphaherpesvirinae, Betaherpesvirinae, and Gammaherpesvirinae. The subfamily Gammaherpesvirinae is comprised of Kaposi’s sarcoma-associated herpesvirus (KSHV), Epstein–Barr virus (EBV), Herpesvirus saimiri (HVS), Rhesus macaque rhadinovirus (RRV), and Murine gammaherpesvirus 68 (MHV68). Several viral encoded genes of the Gammaherpesvirinae subfamily are highly conserved, such as the immediate-early gene ORF50, which encodes the replication and transcription activator RTA [2]. The aim of this review is to highlight what is known about the role of KSHV RTA and its contribution to host-immune evasion through the hijacking of the ubiquitin–proteasome pathway (UPP). Congruently, this review will also touch on the role of other gammaherpesvirus RTA proteins and their role in immunomodulation through the UPP.

KSHV, also known as Human Herpesvirus 8 (HHV-8), was discovered in 1994 and has since been identified as the etiological agent of KSHV Inflammatory Cytokine Syndrome as well as of several cancers and lymphoproliferative diseases such as Kaposi’s Sarcoma, Primary Effusion Lymphoma, and Multicentric Castleman’s disease [3,4,5,6,7]. As with all herpesviruses, KSHV has a biphasic lifecycle—a latent (dormant) state and a lytic (active) phase. During KSHV infection, the UPP is essential for viral entry of KSHV into certain cell types as well as viral egress from infected cells [8]. KSHV will enter the lytic cycle when its RTA protein is expressed. RTA is an immediate-early viral protein that is a potent viral transcription factor, which can directly induce the expression of several cellular and viral genes [9,10,11,12,13]. RTA is necessary and sufficient for the latent-lytic switch of KSHV where all viral lytic genes are expressed in a temporal, cascade-like manner: immediate-early (IE), early (E), and late (L) [2]. In addition to being a potent transcription factor, KSHV RTA has also been shown to ubiquitinate and induce the degradation of proteins through the UPP with its own intrinsic E3 ligase activity, which is mediated by its RING-like domain [14]. This review encompasses what is known about KSHV RTA’s role in host immune evasion through the hijacking of the UPP, and examines whether any other gammaherpesvirus RTA homologs are known to hijack the UPP in a similar function.

## 2. The Ubiquitin–Proteasome Pathway (UPP)

**Figure 1:**
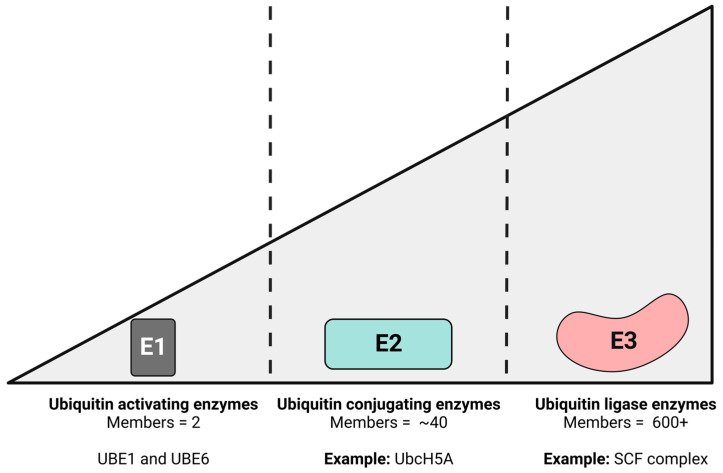
The ubiquitin–proteasome pathway is comprised of three enzymes that function in a temporal cascade leading to cell signaling, protein degradation, DNA repair. The enzymes involved in this cascade are the ubiquitin activating enzyme (E1), ubiquitin conjugating enzyme (E2), and ubiquitin ligase enzyme (E3). The amount of each type of enzyme in depicted above with E1 being the most conserved amount vertebrates while there are several E2 proteins that can transfer ubiquitin to a variety of E3 ligases.

Ubiquitin (Ub) is a reversible, posttranslational modification that can be covalently attached to a substrate protein. Ub is a 76-amino acid polypeptide whose sequence and structure is conserved in all vertebrates [15,16,17]. Cells utilize the UPP to recycle misfolded proteins, for the recycling of membrane bound proteins, the regulation of gene transcription, DNA repair and signaling pathways, and to control the expression of tightly regulated proteins like cell cycle regulatory factors [18,19,20,21,22]. The UPP is composed of multiple enzymes that function in a temporal cascade: E1—ubiquitin activating enzyme, E2—ubiquitin conjugating enzyme (ubiquitin carrier protein), and E3—ubiquitin-protein ligase [23,24,25]. There are only two E1 enzymes, approximately 40 E2 enzymes, and 500–1000 E3 ligases (Figure 1) [26,27,28].

**Figure 2:**
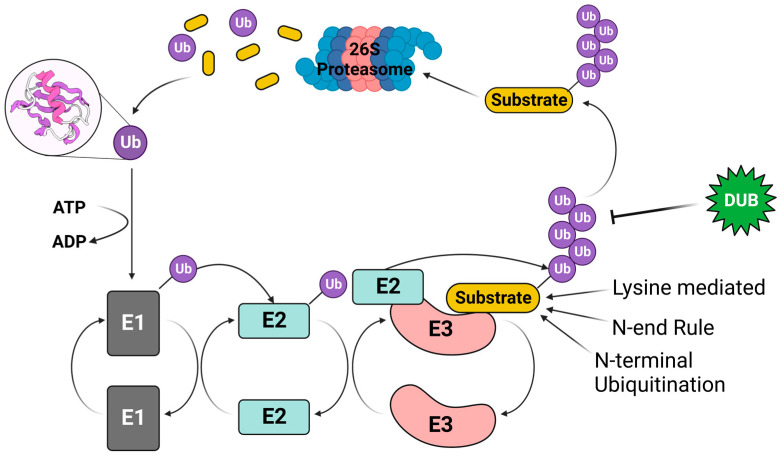
The ubiquitin–proteasome pathway. E1 activates and transfers ubiquitin to E2, the ubiquitin-conjugating enzyme then binds to an E3 ligase, where the ubiquitin is transferred onto the substrate protein. When ubiquitin in linked through K48 chain on a substrate protein it can lead to the degradation of the substrate through the proteasome. However, ubiquitin is a reversible post-translational modification that can be removed by deubiquitinating enzymes (DUBs) to prevent the degradation of a substrate through the UPP.

The E1 activates Ub through ATP hydrolyses causing the formation of a thioester bond between a cysteine in E1′s active site and the carboxyl terminus of Ub [29]. It then transfers the activated Ub to the ubiquitin-conjugating enzyme (E2) using another thioester bond [29,30]. The Ub molecule can then be transferred to the target protein through two different mechanisms by the ubiquitin conjugating enzyme when the E2 interacts with the ubiquitin ligase protein (E3) [27]. The E3 mediates the transfer of Ub to the substrate protein through a covalent isopeptide bond using various mechanisms that involve different E3 proteins with distinct protein domains: (i) E3 can bring the substrate and E2 into close proximity with the target protein to directly transfer Ub to the substrate protein (such as E3 ligases with Really Interesting Gene (RING) domain) or (ii) the E2 transfers the Ub to a cysteine residue of a Homologous to E6AP C-terminus (HECT) domain- or RING-between-RING (RBR) domain-containing E3 ligase that leads to a E3-Ub intermediate before the E3 transfers the Ub to the substrate protein (Figure 2) [31,32].

The addition of polyubiquitin (polyUb) chains, multiple Ub molecules in the form of an isopeptide-linked polymer, to different internal lysine residues of the substrate protein by an E3 ligase can lead to multiple outcomes for the substrate protein such as internalization of a membrane bound protein, activation, cell signaling, or degradation [33,34,35,36]. For example, lysine 48-linked (K48) polyUb chains lead to the proteasomal degradation of the target substrate, whereas K63 polyUb can modulate protein trafficking and signal transduction pathways [37,38]. The sites within each substrate protein in which these PolyUb chains are deposited can differ based on the substrate protein itself, as well as the E2 and E3 enzymes conjugating Ub onto the target proteins [27]. To date, there are six identified amino acid residues/sites where a PolyUb chain can be polymerized from: Lysine, Serine, Threonine, Tyrosine, Cysteine, and the free Methionine residue of the N-terminus [39,40]. Polymerization of a PolyUb chain from an internal lysine residue is the most canonical site for ubiquitin conjugation of proteins targeted for degradation through the UPP. However, proteins can be targeted for degradation through N-terminal ubiquitination, a non-canonical pathway as well (Figure 2). This mechanism is not synonymous to the N-end rule, where an E3 ligase recognizes and binds the N-terminus of a protein containing a degron sequence, which leads to the conjugation of PolyUb chain to an internal lysine residue [41]. In contrast, the N-terminal ubiquitination occurs in a lysine-independent mechanism, where a lysine-48 linked polyUb chain is polymerized from the free N-terminal residue of the target protein (Figure 2) [42].

The covalent binding of ubiquitin molecules to substrate proteins can be reversed by a superfamily of cellular ubiquitin specific proteases called deubiquitinases (DUBs) (Figure 2) [43]. DUBs play an important role in regulating the ubiquitin–proteasome pathway, cell signaling, gene silencing, and the stabilization and subcellular localization of proteins [44,45]. During the co-evolutionary adaptation to their hosts, viruses additionally evolved to utilize both the UPP and DUB pathways to modulate the function of cellular and viral proteins in infected cells with the goal of resisting detection by the hosts immune system in order to enable reproduction [46].

## 3. KSHV-Encoded Viral Factors Inducing Protein Degradation

**Figure 3:**
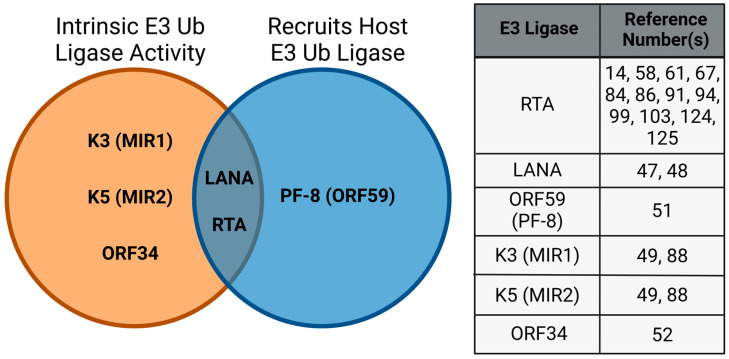
KSHV encodes seven proteins that contain intrinsic E3 ubiquitin ligase activity and/or recruit host E3 ubiquitin ligases to ubiquitinate and induce the substrates degradation through the UPP.

KSHV encodes for several viral proteins to target host or viral proteins for degradation such as Latency Associated Nuclear Antigen (LANA), K3 (MIR1), K5 (MIR2), Processivity Factor-8 (PF-8/ORF59), ORF34, and RTA [14,47,48,49,50,51,52]. These viral proteins either contain intrinsic E3 ubiquitin ligase activity themselves, or hijack and recruit a host E3 ubiquitin ligase to degrade a target substrate protein; some viral proteins have the capacity to do both, depending on the cellular context (Figure 3). These viral factors are important for immune modulation, the promotion of viral maintenance, or virus production, and the majority of their roles have been previously discussed in other reviews [53,54]. This review will focus on what is currently known about the role of KSHV RTA as an E3 ubiquitin ligase and its homologs within the gammaherpesvirus subfamily.

RTA and its transactivating function have been studied since 1998; however, its E3 ligase activity was not identified until 2005 [14,55]. While all of RTA’s functions are fundamental for the survival and dissemination of KSHV, the goal here is to highlight the importance of RTA’s bimodal activity in targeting proteins for degradation through using its own E3 ligase activity or by stabilizing and chaperoning host E3 ligases. Since the discovery of RTA’s E3 ligase activity, literature has shown that RTA preferentially targets sumoylated proteins for proteasomal degradation through the UPP [56]. This function of RTA helps to subvert the host’s innate and adaptive immune responses while also modulating the host transcriptome and protein landscape to promote virus production.

## 4. RTA Suppresses Innate and Adaptive Immune Responses Induced by KSHV Infection

The innate immune system is the host’s first line of defense against viruses, which can be triggered during both primary KSHV infection and viral reactivation from latency. The innate immune system is comprised of several pattern-recognition receptors (PRRs) that can detect foreign materials produced from pathogens called pathogen-associated molecular patterns (PAMPs). In this regard, adaptor proteins such as MyD88, TRIF and TRAM play a crucial role to initiate a cascade of several signaling molecules downstream of the PRRs leading to the activation of NF-κB and IRFs ultimately resulting in the production of type I IFN and various proinflammatory cytokines [57]. RTA is also known to inhibit pathways of the host’s immune responses [14,58]. In fact, 50% of the proteins that RTA is known to target for degradation are instrumental in the regulation of innate and adaptive immune system. RTA blocks innate immune responses mediated by TLR3 and TLR4 signal transduction and type I IFN induction through targeting TRIF, MyD88, IRF3, and IRF7.

### 4.1. Toll-Interleukin-1 Receptor Domain-Containing Adaptor Protein-Inducing Interferon β (TRIF)

TRIF, an adaptor protein in the TLR signaling pathway, is necessary to mediate signal transduction from TLR3 and TLR4 to IRF3, resulting in type I IFN production [59,60]. KSHV RTA was shown to mediate the degradation of TRIF through the ubiquitin–proteasome pathway as another mechanism to suppress host innate immunity [61]. It was later found that the activation of the TLR3-TRIF pathway enhanced the expression of RTA through increasing the translation efficiency of RTA mRNA [62]. In the context of KSHV infection, it is believed that this feedback loop is activated during KSHV infection where the TRL3-TRIF pathway is stimulated and increases the amount of RTA protein expression, which, in turn, degrades TRIF to dampen type I IFN production [62].

### 4.2. Myeloid Differentiation Factor 88 (MyD88)

MyD88 is a multifunctional adaptor protein that is essential for mediating cell signaling initiated by interleukin-1 (IL-1), IL-18, and IL-33 receptors and all TLR signaling except for TLR3 [63,64,65]. As such, MyD88 is integral to innate and adaptive immunity [66]. During KSHV lytic reactivation and de novo infection, KSHV RTA has been shown to mediate the degradation of MyD88 through the ubiquitin proteosome pathway using its E3 ubiquitin ligase activity by direct interaction and poly-ubiquitination of this adaptor protein [67]. Thus, KSHV RTA is equipped to dampen innate and adaptive immune responses by targeting MyD88 and TRIF, two vital adaptor proteins for receptor-mediated signal transduction, for degradation through the ubiquitin–proteasome pathway.

### 4.3. Interferon Regulatory Factors 3 and 7 (IRF3 and IRF7)

Type I interferon (IFN), IFN-α and -β are activated during viral infection and can block viral replication through several different mechanisms that are employed by the vast range of IFN-induced gene products [68]. Interferon regulatory factors (IRFs), more specifically, IRF1, IRF3, IRF5, and IRF7, are important for the induction of IFNs and Interferon-stimulated genes (ISGs) during viral infection [68,69]. As with other viruses, KSHV encodes for several viral proteins to counteract the IFN signaling pathway, such as viral interferon regulatory factors (vIRFs), ORF10, ORF45, K8, ORF52, LANA, and RTA [67,70,71,72,73,74,75,76,77]. It was first shown that RTA itself can bind and degrade IRF7 via the ubiquitin–proteasome pathway using its own E3 ubiquitin ligase activity through its RING-like domain [14]. This mechanism leads to the attenuation of type I IFN production, and its antiviral affect during KSHV de novo infection and reactivation [14]. The same group later found that in addition to RTA’s direct interaction with IRF7, KSHV RTA also stabilizes and recruites the cellular HECT E3 ubiquitin ligase RAUL (RTA-associated ubiquitin ligase, also known as KIAA10 or UBE3C) to target both IRF3 and IRF7 for degradation through the ubiquitin–proteasome pathway [58]. RTA was shown to bind, stabilize, and enhance RAULs E3 ligase activity towards IRF3 and IRF7 by recruiting host deubiquitinating enzyme, USP7, to further stabilize RAUL [58]. Taken together, this shows that RTA hijacks USP7 to bind and sustain RAUL expression, leading to an enhanced suppression of IRF3, IRF7, and type I IFN production and antiviral activity.

### 4.4. Signal Transducer and Activator of Transcription 6 (STAT6)

STAT6, like all seven members of the STAT family, are transcription factors that are activated through cytokines and growth factors to mediate signal transduction from the plasma membrane to the nucleus [78]. STAT6 is vital for both innate and adaptive immune responses. While STAT6 can be stimulated by interleukins 3 and 15 (IL-3 and IL-15), growth factors, and interferon alpha, it is primarily activated by IL-4 and IL-13 [79]. A unique characteristic of STAT6 is that it can be activated in a JAK-independent manner during viral infection, which leads to the production of the chemokine CCL2 to recruit T cells to the site of infection [80]. Additionally, STAT6 plays a role in the maturation and proliferation of B cells, induces the expression of MHC class II, and promotes immunoglobulin class switching to IgE and IgG1 [79]. KSHV has been shown to hijack STAT6 activation and subcellular localization to promote cell survival and viral latency [81,82,83]. During KSHV latency, it has additionally been shown that LANA induces the cleavage of STAT6 so that it acts as a dominant negative regulator of the transcription to RTA, thus ablating the entire lytic cycle [81]. It was recently discovered that during KSHV lytic reactivation, STAT6 is downregulated at the protein level and not at the RNA level in PEL, iSLK, and endothelial cells [84]. It was determined that KSHV RTA rapidly polyubiquitinated STAT6 through K48 and K63 linked chains leading to the induction of its proteasome- and lysosome-mediated degradation [84]. KSHV RTA’s E3 ligase activity in its RING-like domain was also required to mediate the ubiquitination of STAT6. The degradation of STAT6 by KSHV allowed for the cellular E3 ligase TRIML2 to by ubiquitinated to prolong cell survival and a robust lytic cycle [84]. Overall, Weng et al., determined that STAT6 must be degraded and TRIML2 must be ubiquitinated for KSHV RTA to be robustly expressed leading to a potent activation of the KSHV lytic cycle [84]. They also determined that EBV, HCMV and HSV-1 lytic replication leads to the degradation of STAT6 and the ubiquitination of TRIM2L suggesting that the IE proteins from each of these viruses may also play a role in the degradation of STAT6 and ubiquitination of TRIML2 [84]. Further studies will need to be conducted to determine if there is a conserved interplay between the IE proteins of each human herpesvirus subfamily from Herpesviridae and STAT6 or if this is a compounding affect from several viral factors in certain herpesvirus subfamilies.

### 4.5. Major Histocompatibility Complex, Class II, DR Alpha (HLA-DRα)

HLA-DRα is a member of the MHC-II protein family and has an important role in the adaptive immune response by presenting cellular and viral antigens to CD4 T helper cells [85]. It is important for KSHV to evade this mechanism to ensure the survival of infected cells in the host. RTA was shown to directly bind and promote the degradation of HLA-DRα [86]. It was also determined that in addition to RTA directly inducing HLA-DRα’s degradation, it can also upregulate the expression of the cellular E3 ligase, MARCH8, to promote the internalization and degradation of HLA-DRα through the ubiquitin–proteasome pathway [86,87]. In addition to RTA targeting MHC class II molecule for degradation, KSHV also encodes for two additional E3 ligases, K3 and K5, to mediate the protein degradation of MHC class I molecules [49,88]. RTA working in concert with these two other viral E3 ligases KSHV can robustly repress the adaptive immune response towards infected cells.

## 5. RTA-Mediated Degradation of Cellular Repressors of RTA

After de novo infection of KSHV, in most cell types, the default state of the KSHV is latency. During latency, several cellular factors are upregulated to promote viral maintenance by repressing lytic gene induction. In turn, during KSHV reactivation, RTA is now known to target a couple of these factors for degradation to promote its own expression and consequently the entire viral lytic cycle.

### 5.1. Inhibitor of DNA Binding Protein (ID) Family

All four members of the ID protein family, ID1-ID4, are helix-loop-helix proteins and potent transcriptional repressors [89]. In 2014, it was shown that LANA upregulates ID1, 2, and 3 in KS lesion through the BMP-Smad1 pathway and they are involved in KSHV-induced tumorigenesis [90]. Our group has recently shown that RTA induces the degradation of ID2 during lytic reactivation of KSHV in primary effusion lymphoma (PEL) cells. We demonstrated that RTA interacts with ID2 and polyubiquitinates it through N-terminal ubiquitination to induce the degradation of ID2 through the UPP in order to promote its own expression and virus production [91]. To our knowledge, this was the first study to demonstrate that KSHV RTA can induce the N-terminal ubiquitination of a substrate protein. We also interrogated whether the ability of KSHV RTA to target ID2 for degradation through the UPP was evolutionarily conserved between other RTA proteins from Gammaherpesvirinae and the members of the ID protein family. We determined this interplay between the Gammaherpesvirus RTAs from KSHV, EBV, MHV68, and all members of the ID protein family was indeed conserved by showing that each ID protein was robustly reduced in the presence of each gammaherpesviral RTA [91]. Additional studies will be needed to further elucidate the exact mechanism of how KSHV, EBV, and MHV68 RTA degrades each ID protein as well as its importance for the regulation of the life cycle of different gammaherpeviruses.

### 5.2. Hairy/Enhancer-of-Split Related with YRPW Motif Protein 1 (Hey1)

During KSHV latency, LANA promotes KS angiogenesis by activating the Notch signaling pathway and hijacking Hey1, a downstream target of Notch [92]. Hey1 is a basic helix-loop-helix (bHLH) transcription repressor. By homo-/hetero-dimerizing with other bHLH proteins, Hey1 will bind a target promoter and recruit corepressors such as mSin3A [93]. RTA was shown to induce Hey1 expression while Hey1 can repress the RTA promoter, which results in the maintenance of latency [94]. Another study found that, RTA induces the degradation of Hey1 through the UPP during KSHV reactivation in PEL cells [94,95]. When considering both studies, their findings coincide with the timepoints interrogated during the lytic reactivation of KSHV, since Gould et al. explored the interplay between Hey1 and RTA within the first 24 h of KSHV reactivation while Yada et al. looked at or after 24 h [94,95]. Taking these two observations together, there seems to be a cyclic feedback loop between RTA and Hey1. While RTA initially depletes Hey1 by targeting it for degradation through the UPP up to 24 h post-reactivation to allow for the robust induction of RTA, Hey1′s mRNA abundance eventually increases, and its protein level rebounds to suppress RTA expression allowing for the lytic cycle to progress through the early and late stages of reactivation [94,95]. This exchange by RTA with Hey1 subtly mimics the interplay between RTA and the cellular repressor K-RBP.

### 5.3. KSHV-RTA Binding Protein (K-RBP)

K-RBP, also known as MGC2663 or ZNF426, is a Kruppel-associated box (KRAB)-containing zinc finger protein, which was discovered to interact with KSHV RTA in the yeast two-hybrid system [96]. It was demonstrated that K-RBP binds RTA and acts synergistically with it to enhance the transactivation of several of RTA’s target genes such as ORF57, K8, vMIP-I (aka K6), and RTA itself [96]. However, it was later determined that while K-RBP does promote RTA transactivation, it is only when K-RBP is at lower concentrations [97,98]. When K-RBP is more abundant in the cell, it acts as a transcriptional repressor even in the context of KSHV infection [97,98]. After K-RBP’s repressive function was revealed, it was determined that during KSHV reactivation, RTA itself promoted the degradation of K-RBP through the UPP using its RING-finger like domain to promote the lytic cycle of KSHV [99].

### 5.4. Structural Maintenance of Chromosome 5 and 6 (SMC5/6) Complex

The SMC5/6 complex is one of four chromosome maintenance complexes that share core characteristics: a pair of SMC ATPases hinge to bring their N- and C-terminal together to form a ring structure that contains ATPase and DNA-binding activity [100]. There are six additional members in the SMC5/6 complex; they are called non-SMC elements (NSEs), and each cofactor plays an important role in the structure and function of the complex [100,101]. The SMC5/6 complex plays an important role in chromosomal DNA repair, such as double-stranded breaks, and it is also involved in replication fork stability [100]. This complex has also been shown to further compact the DNA through its ATPase activity [100]. The SMC5/6 complex has been shown to repress the replication of DNA viruses through epigenetic silencing, such as Hepatitis B virus, unintegrated viral DNA of Human Immunodeficiency Virus (HIV), Human papillomavirus (HPV), Epstein–Barr virus (EBV), and Kaposi’s Sarcoma-associated Herpesvirus (KSHV) [102]. Recently, it was determined by Han et al. 2022 that the SMC5/6 complex inhibits the lytic replication of KSHV when overexpressed, while the knockdown of the complex promotes lytic replication of KSHV [103]. Additionally, they determined that the SMC5/6 complex suppressed the lytic cycle of KSHV by removing H3K27ac from the KSHV chromatin in a ATPase and DNA-binding dependent mechanism to cause further condensation of the viral episome [103]. However, they found that during reactivation of the virus KSHV RTA targets the SMC5/6 complex for ubiquitination and subsequent degradation through the UPP [103]. Interestingly, they determined that the RTA proteins from Epstein–Barr virus (EBV), nonhuman primate rhesus Rhadinovirus (RRV), herpesvirus saimiri (HVS), and murine γ-herpesvirus 68 (MHV68) can also downregulate SMC5 and SMC6 suggesting an evolutionarily conserved interaction between the γ-herpesvirus RTAs and the SMC5/6 complex [103].

## 6. Viral Targets of KSHV RTA for Protein Degradation

### 6.1. K-bZIP (K8)

K8 is an early viral protein induced upon KSHV reactivation [104]. Unlike RTA, K8 is not sufficient for the latent-lytic switch of KSHV; however, it is instrumental for viral replication to occur during de novo infection [105]. K8 is responsible for binding to ori-Lyt and recruiting the viral and cellular factors that make up the viral replication complex in conjunction with RTA [106,107]. It has been shown by several groups that K8 binds to RTA in reactivated cells to selectively repress RTA transactivation activity towards certain viral promoters and repress the viral lytic cycle when over-expressed during reactivation [108,109]. There is currently only one study that has demonstrated that RTA causes the degradation of K8 through the UPP [99]. Further studies will be needed to determine the exact mechanism by which RTA targets K8, what the cellular context is for K-bZIP degradation, and why K8 is targeted. However, based on the literature as a whole, it seems that RTA ablates the expression of K8 to further promote the lytic cycle since studies have shown that RTA overexpression can compensate for the loss of K8 to progress through the lytic cycle [110].

### 6.2. Latency-Associated Nuclear Antigen (LANA)

LANA encoded by the viral gene ORF73 is an essential latent viral protein necessary for viral episome maintenance, as well as host immunomodulation, during KSHV latency (reviewed in [111]). In addition to this, LANA regulates specific viral and host factors to promote latency. LANA antagonizes RTA’s expression in several different ways. For example, LANA represses RTA expression by recruiting KAP1 onto the promoter of ORF50 and by binding RBP-Jκ, a known activator and co-factor of RTA, to block the Notch signaling pathway while also sequestering it away from RTA’s promoter [112,113,114,115,116,117]. It has been shown that LANA is targeted by RTA for proteasomal degradation when they are co-transfected into cells [99]. Further studies are needed to elucidate the exact mechanism of the RTA-mediated degradation of LANA and to determine if RTA can target LANA for protein degradation in KSHV-infected cells as well. LANA and all other latent proteins are constitutively expressed and are essential for viral maintenance so even though RTA does target LANA for proteasomal degradation, its protein level is not fully abolished. Instead, it seems that RTA reduces its abundance to a certain level that is permissive for robust KSHV lytic reactivation [99]. Further studies are needed to elucidate whether RTA’s E3 ligase activity is necessary for LANA’s degradation or if it is mediated through a different mechanism.

### 6.3. vFLIP

The viral FLICE inhibitory protein (vFLIP) encoded by ORF71 is constitutively expressed during KSHV latency similar to LANA, and it promotes KSHV latency by inhibiting viral lytic replication [118]. vFLIP interacts with and activates the IκB Kinase complex and subsequently leads to the induction of the NFκB pathway which in turn inhibits lytic gene activation [119,120,121]. The vFLIP-mediated stimulation of this pathway has been shown to be necessary for the maintaining of viral latency in promoting cell survival [122,123]. RTA has been shown to mediate the degradation of vFLIP through the UPP by recruiting host E3 ubiquitin ligase, ITCH [124,125]. This interaction results in the downregulation of NFκB signaling, a reduction of TNFα production, and an induction of lytic gene expression [124,125].

## 7. Gammaherpesvirus RTA Homologs

**Figure 4:**
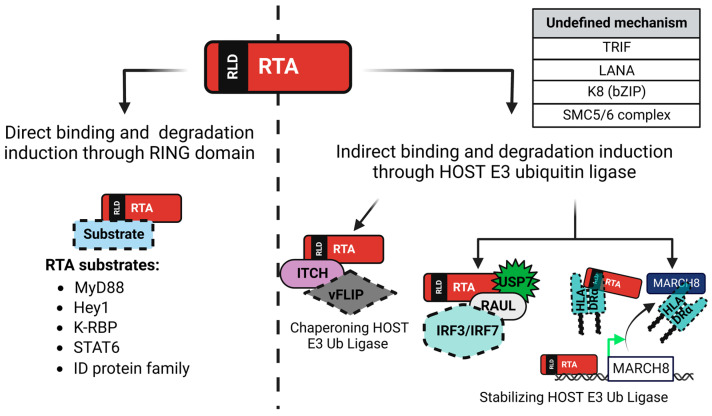
KSHV RTA has an intrinsic E3 ligase activity and the capacity to stabilize and chaperone a host E3 ligase to target its substrate proteins that fit into two distinct categories. The first set of RTA’s substrate proteins are the transcription repressors that suppress the viral lytic cycle such as ID2, Hey1, K-RBP, and K8 or indirectly lead to the repression of RTA’s function and induction of RTA expression like LANA. The second set of proteins targeted by KSHV RTA are cellular and viral factors that mediate innate immune signaling such as vFLIP, TRIF, MyD88, IRF3, and IRF7 or adaptive immune presentation like HLA-DRα. RLD: RING-like domain.

KSHV RTA targets both cellular and viral proteins for proteasomal degradation to promote the lytic cycle of KSHV (Figure 4). By targeting these cellular and viral factors KSHV RTA establishes an environment that promotes efficient virus production without alerting the hosts immune cells and being eradicated. Because KSHV RTA has homologs in other gammaherpesviruses that all have similar transactivation functions, we looked to see if other studies had shown that these RTA homologs could also target specific cellular and viral proteins for degradation through the UPP [126,127].

### 7.1. The Lymphocryptovirus: Epstein–Barr Virus (EBV)

EBV is a lymphocryptovirus that can cause infectious mononucleosis, Burkitt lymphoma, nasopharyngeal carcinoma, Hodgkin’s lymphoma, and other cancers [128]. In EBV, Rta upregulates the expression of Zta to induce reactivation from latency, however, it does not play the same sole master regulatory role as KSHV’s RTA [129,130]. In addition to being a transactivator protein, EBV Rta has immunomodulatory effects by negatively regulating the promoter activity of IFN-β as well as IRF3 and IRF7 endogenous expression at both the protein and RNA level [131]. It was also demonstrated, through co-transfection in HEK293T cells, that expression of FLAG-tagged IRF1, IRF2, IRF3, and IRF7 were abolished in the presence of Rta [131]. Although this group did not use proteasome inhibitors to see if IRF1, IRF3 and IRF7 protein levels could be restored in the presence of RTA, this data infers that EBV Rta is inducing the downregulation of these IRFs at the protein level [131]. An additional study identified that EBV Rta can induce the downregulation of the SMC5/6 complex at the protein level although they also did not use a proteasome inhibitor to test if EBV Rta degraded the complex through the proteasome pathway [103]. Since KSHV and other IE proteins from other herpesvirus subfamilies have been shown to induce the degradation of IRFs, it is possible that EBV Rta may also have this ability even though it was not explicitly indicated in this study [131]. However, another study demonstrated that EBV Rta can induce the degradation of SUMOylated proteins in a proteasome-dependent manner, which resembles to the activity of KSHV RTA [56,132]

### 7.2. The Rhadinoviruses: Herpesvirus Saimiri (HVS), Rhesus Monkey Rhadinovirus (RRV), and Murine Gammaherpesvirus 68 (MHV-68)

Herpesvirus Saimiri (HVS) is a gammaherpesvirus that causes persistent infection of a subset of T lymphocytes in squirrel monkeys (*Saimiri sciureus*) and is part of the *Rhadinovirus* genus [133,134]. While HVS does not cause maladies in its natural host, squirrel monkeys, it has been shown to cause acute T cell lymphomas in several New World primate species such as cinnamon ringtail monkeys, common marmosets, and owl monkeys [135,136,137]. As with all gamma-herpesviruses, HVS encodes an ORF50 gene, also referred to as EcoRI-D or HVS R protein, and shares significant sequence homology to EBV Rta and induces the reactivation from latency [138,139,140]. However, to date, no studies have determined if HVS R protein contains any intrinsic E3 ligase activity or can stabilize and chaperone a host E3 ligase. However, Han et al., identified that HVS R protein can downregulation the SMC5/6 complex at the protein [103]. Additional studies are needed to determine if the HVS R protein contains intrinsic E3 ligase activity.

Rhesus monkey Rhadinovirus (RRV) was discovered in 1997 and was found to be very closely related to KSHV in both genome organization and sequence [141,142,143,144,145]. RRV lineage 1 (RV1), the most highly related lineage to KSHV, causes similar disease in rhesus macaques that KSHV does in humans. Co-infection of rhesus macaques with simian immunodeficiency virus (SIV), the equivalent to human immunodeficiency virus (HIV) in humans, and RRV leads to the development of B cell hyperplasia lymphoproliferative diseases that are seen in human AIDS patients that are coinfected with KSHV [146,147,148,149]. While both RRV and KSHV RTAs have been shown to have high transactivation activity and have high sequence homology in their N-terminus, there are no studies that have looked at the potential E3 ubiquitin ligase activity of RRV RTA [150,151,152]. The sequence homology of RRV RTA in the N-terminus, where the RING-like domain of KSHV RTA is located, suggest that there is potential E3 ligase activity of RRV RTA that may be an untapped area in the field of RRV virology. It is possible that there may be divergence in the type of targets RRV RTA may have from KSHV RTA’s E3 ligase activity, since we discovered that RRV RTA was unable to cause the downregulation of the ID protein family, while KSHV, EBV, and MHV68 RTA could [91]. There is, however, one study that demonstrated that RRV RTA can induce the downregulation of the SMC5/6 complex at the protein level. Although this group did not use a proteasome inhibitor to test if EBV Rta degraded the complex through the proteasome pathway, this group’s evidence suggests that RRV RTA might have intrinsic E3 ligase activity [103].

Murine gammaherpesvirus 68 (MHV-68), also known as Murid Herpesvirus-4 and γHV68, naturally infects small, wild rodents and can cause pathogenesis in inbred and outbred mice [153,154,155]. Unlike other gamma-herpesviruses, initial MHV68 infection can occur intranasally where it travels to the lung for up to a week before causing splenomegaly and establishing latency in activated/cycling B cells [156,157,158,159,160]. Similar to EBV and KSHV RTA, MHV-68 RTA plays a critical role in viral reactivation from latency and the expression of its viral early and late genes during lytic replication [161,162]. To our knowledge, there has been just one group that has demonstrated the E3 ligase activity of MHV68 RTA where they discovered that RTA contains E3 ligase activity, which allows it to ubiquitinate and induce the degradation of RelA through the ubiquitin–proteasome pathway to suppress NFκB activation and cytokine production [163]. However, there is another group that has implied that MHV68 RTA may also induce the degradation of SMC5/6 complex [103].

## 8. Structural Comparison of Gammaherpesvirus RTAs

Gammaherpesvirus studies have currently determined that the RTA encoded by KSHV and MHV68 contain E3 ligase activity while EBV Rta’s putative E3 ligase activity has been inferred but not determined [132,163]. There are no current studies indicating that HVS or RRV encoded RTAs have intrinsic E3 ligase activity. There are several different types of E3 ubiquitin ligases, but the type of E3 ligase domain that KSHV RTA resembles most is a RING E3, because they are cysteine rich [14,164]. RING E3 ligases are known to coordinate two zinc ions in their alphahelical domains [164]. KSHV RTA is not considered a RING E3 ligase because it does not meet all the criteria, but is considered RING-like because of its Cys/His rich-region from amino acid positions 118–207 [14,54]. Further studies interrogating whether EBV, RRV, and HVS RTA also contain ubiquitin ligase activity would be instrumental to the field of gammaherpesvirology since this could suggest evolutionary conservation in the types of substrate proteins that these gammaherpesvirus RTA proteins target for degradation. For example, our group recently published findings suggesting this phenomena with MHV68, EBV, and KSHV RTA since they were all able to downregulate protein level of all the ID protein family members [91].

**Figure 5:**
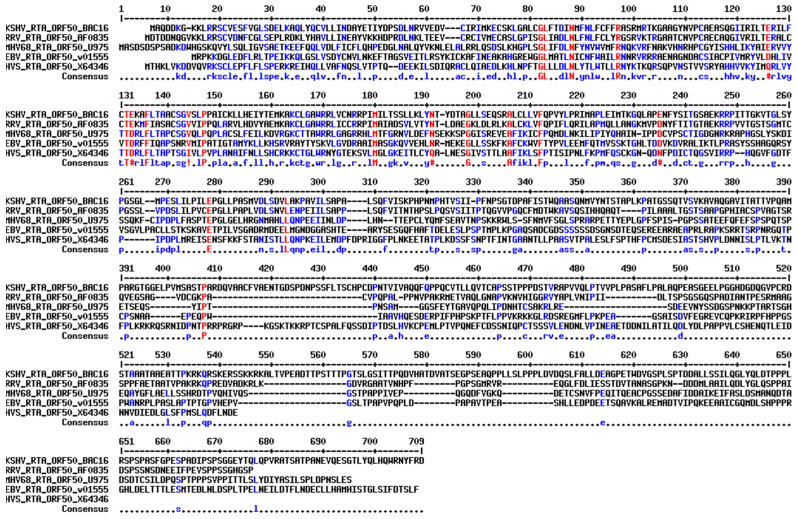
Amino acid sequence alignment between KSHV, EBV, MHV68, RRV, and HVS encoded RTA. Red—High consensus (90%), Blue—low consensus (50%), **#**—if there is N, D, Q, or E majority consensus, **!**—if there is a I or V majority consensus. Software used: Multalin.

**Figure 6:**
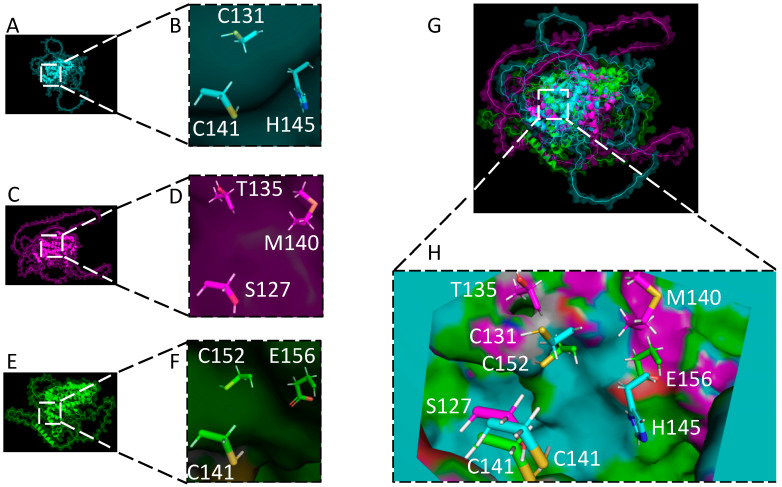
The predicted RTA protein structure of KSHV (blue), EBV (magenta), and MHV68 (green) are depicted in panels (**A**,**C**,**E**) respectively. Additionally, zoom in views of the three gammaherpesvirus RTAs are respectively shown in panels (**B**,**D**,**F**) to highlight their three amino acids that may possibly be involved in zinc coordination. A superimposition of the three herpesvirus RTAs are shown in panel (**G**) with a zoom in view in panel (**H**) highlighting the conserved interior pocket observed in all three herpesvirus RTAs.

The alignment of the amino acid sequences of all the gammaherpesvirus RTAs implies a slight homology in their N-terminal region where KSHV RTA’s RING-like domain is found (Figure 5). To further examine this sequence, we used the protein structure prediction software AlphaFold to determine if EBV RTA structurally contains the E3 ligase domain of KSHV and MHV68 in the same region based on the predicted folding of each RTA protein [165]. The resulting structures were then superimposed using the software program Coot and figures were made in PyMOL (version 0.9.4; Schrodinger) for visualization (Figure 6) [166]. Figure 6 shows the structures of the RTA protein of KSHV (blue) in A, EBV (magenta) in C, and MHV68 (green) in E, with corresponding close-up views in B, D, and F, respectively. The views are oriented around the RTA residues C131, C141, and H145 of KSHV, which have been implicated in the E3 ligase activity of KSHV RTA [14,67,94]. Additionally, residues C141 and C152 in MHV68 have also been shown in the literature to be required for E3 ligase activity [163]. While E156 of MHV68 has not been shown, this residue is in the same location as KSHV H145 when superimposed, suggesting it could play a role in the coordination of a zinc atom. EBV has yet to be shown that it contains E3 ligase activity, but based on the structural modeling we propose that residues S127, T135, and M140 could act as such a catalytic active site. In fact, serine, threonine, and methionine residues, even though they are weaker co-factors, have previously been shown to coordinate zinc [167,168,169]. Additionally, the three amino acids of EBV occupy the same special positioning as the three residues observed in KSHV, suggesting a conserved function. 

A superimposition of KSHV, EBV, and MHV68 RTAs is represented in Figure 6G. Overall, this superimposition of atomic coordinates which minimized the global Root Mean Square Deviation (RMSD) of these three RTAs are between 26–39 Å when comparing any two of the RTA proteins. At first glance, this high RMSD would indicate no structural homology, with variability across the entire family of proteins (Figure 6G), but when only considering a conserved interior pocket that is present in all three proteins (Figure 6H) the RMSD for the alpha helical core (residues 1–340) of KSHV and the alpha helical core (residues 1–340) of MHV68 is 1.3 Å; the core of KSHV and the alpha helical core (amino acids 1–255) of EBV is 1.5 Å; and the cores of MHV68 and EBV is 1.2 Å. Hence, with these three low RMSD values, the models indicate a conserved structural pocket exists within the three RTA proteins.

This structural conservation is significant, specifically as C141 of KSHV, S127 in EBV, and C141 of MHV68 are structurally invariant, with an RMSD 1.7 Å between side-chain sulfur and oxygen atoms of respective amino acids. While the other two binding sites in the pocket are less conserved, they are similar enough between the viruses to suggest a conserved structure motif for a zinc-binding site. The C131 of KSHV, T135 of EBV, and C152 of MHV68 are between 6.5 and 8.1 Å from one another’s side-chain oxygen or sulfur while H145 of KSHV, M140 of EBV, and E156 of MHV68 are between 7.8 and 10.6 Å apart from corresponding nitrogen, oxygen, and sulfur atoms in the residue side-chains of compared RTAs. These three amino acids between the three viruses add to the hypothesis of a conserved structure and function of RTA across the family of gammaherpesviruses for binding zinc that corresponds to the E3 ligase activity. 

It was previously reported that KSHV RTA has a RING-like domain from residues 118–207 [14]. In this stretch, there are four additional cysteines at positions 121, 155, 164, and 196. Interestingly, in the predicted structures all four of these amino acids are located within the same pocket observed in Figure 6B. As such, these may also be key functional residues in E3 ligase activity that should be explored further. RING E3 ligases coordinate two zinc ions in a “crossbrace” arrangement to allow for E2 binding and to catalyze the addition of ubiquitin onto a substrate protein [170]. Since KSHV RTA contains a RING-like domain, it is possible it can coordinate two zinc ions within its pocket with eight residues that could be a combination of those described here or other known metal coordinating amino acids, such as histidine, threonine, methionine.

## 9. Conclusions

KSHV encodes several, multifunctional viral proteins to modulate the hosts immune response and to create an environment conducive for viral maintenance and production by hijacking the UPP and causing the degradation of several cellular and viral proteins. Some examples of these viral factors are LANA, K3, K5, PF-8, ORF34, and RTA (Figure 3). A growing body of evidence shows that RTA is more than just a transcription activator protein. This review focused on how KSHV RTA targets several cellular and viral factors to allow for robust lytic reactivation. The substrate proteins of KSHV RTA fit into two broad categories. They either repress transcription of viral genes, even the RTA promoter itself, or they are involved in restriction of virus production by triggering the innate and adaptive immune response (Figure 4). Because KSHV belongs to the Gammaherpesvirinae subfamily and each virus in this subfamily encodes for an RTA homolog, we were interested in whether any groups have previously shown that these RTA homologs have E3 ligase activity similar to KSHV RTA. Using, in silico structural alignment and folding predictions, we demonstrated that C131, C141, and H145 in KSHV RTA coincide with C141 and C152 in MHV68 RTA (Figure 6). While EBV Rta has not been experimentally shown to contain E3 ligase activity, it does possess equivalent catalytic amino acids that KSHV and MHV68 encoded RTAs have. Remarkably, the RTAs from KSHV, MHV68, and EBV all contain a pocket surrounding these three key amino acid residues where we hypothesize zinc could be coordinated. Additional enzymatic and crystallographic experiments will be needed to elucidate the significance of these findings and how they contribute to the function of gammaherpesviral RTAs.
